# Effective adsorption of heavy metal ions in water by sulfhydryl modified nano titanium dioxide

**DOI:** 10.3389/fchem.2022.1072139

**Published:** 2023-01-27

**Authors:** Beibei Chen, Lin Li, Lei Liu, Jianxin Cao

**Affiliations:** ^1^ College of Chemistry and Chemical Engineering, Guizhou University, Guiyang, China; ^2^ Guizhou Academy of Testing and Analysis, Guiyang, China

**Keywords:** adsorption, hybrid nanocomposite, sulfhydryl modified, heavy metals, SERS

## Abstract

**Background:** The monitoring and removal of heavy metal ions in wastewater will effectively improve the quality of water and promote the green and sustainable development of ecological environment. Using more efficient adsorption materials and more accurate detection means to treat heavy metal ions in water has always been a research focus and target of researchers.

**Method:** A novel titania nanomaterial was modified with sulfhydryl group (nano TiO_2_-SH) for detection and adsorption of heavy metal ions in water, and accurately characterize the adsorption process using Surface-Enhanced Raman Spectroscopy (SERS) and other effective testing methods.

**Results:** The maximum adsorption efficiency of nano TiO_2_-SH for the Hg^2+^, Cd^2+^, Pb^2+^ three heavy metal ions reached 98.3%, 98.4% and 98.4% respectively. And more importantly, after five cycles of adsorption and desorption, the adsorption efficiency of nano TiO_2_-SH for these three metal ions is still above 96%.

**Conclusion:** These results proved the nano TiO_2_-SH adsorbent has great potential in practical water pollution purification.

## Introduction

With the rapid industrial development, the pressure on water ecological environment is also increasing. Heavy metal ions pollution in water is mainly caused by the cleaning and discharge of materials in industrial production processes, such as pickling wastewater from metal smelting, crushing and leaching of ore mining, lead-acid battery manufacturing, leather tanning treatment, and synthesis of related chemical products. (Ma et al., 2020a; Ma et al., 2020b; Li et al., 2021; Yan et al., 2021; He et al., 2022). The sewage of heavy metals cannot be easily biodegraded, which causes serious deterioration in the ecological environment; the damage is mainly reflected in the accumulation of these metals in the human organs that may lead to intellectual disability, developmental delay, liver cirrhosis, and kidney exhaustion. (Bolisetty et al., 2019; Shao et al., 2022; Singha et al., 2022). Therefore, highly efficient methods for separation and enrichment of heavy metal ions are urgently required.

Over the last few decades, several strategies for removing heavy metal ions, including flotation, chemical precipitation, adsorption, membrane separation, ion exchange, electrodialysis, and biological processes. (Wu et al., 2019; Hasanpour and Hatami, 2020; Wang et al., 2020; Gupta et al., 2021). Among these methods, the use of adsorption is advantageous in practical applications because of the high efficiency, simple operation, reusability, and low cost associated with this approach. The adsorbents are roughly divided into five types: modified silicon-based materials, modified aluminum-based materials, modified manganese-based materials, modified carbon-based materials, and biological materials. (Choi et al., 2020; Xie et al., 2021; Chen et al., 2022; Shi et al., 2022; Xu et al., 2022). In recent years, nano titanium dioxide (nano TiO_2_) materials have been used as adsorbent materials by many researchers due to their advantages of large specific surface area and porosity. The modification of nano TiO_2_ can effectively improve its adsorption efficiency for more kinds of materials through the interface assembly of organic components. (Dong et al., 2017; Huang et al., 2019; Zhou et al., 2019; Qiang et al., 2020; Roy, 2022).

On the other hand, Surface-enhanced Raman spectroscopy (SERS) has become a rapid detection method with great potential developed in recent years due to its advantages, including fast detection, simple operation, simple equipment, high sensitivity, *etc.* (Grasseschi and Toma, 2017; Sun et al., 2019) As a detection technology to obtain the characteristic information of trace substances by inelastic scattering fingerprint signals of molecules and the enhancement of precious metal substrate, SERS technology has shown unique application advantages and prospects in the fields of materials, medicine, food, safety, *etc.* (Cardinal et al., 2017; Sahin et al., 2022) The heavy metal ions (such as: Hg^2+^, Cd^2+^, Pb^2+^) concentration in environmental water is low and the matrix is complex, so it is difficult for conventional detection instruments to obtain ideal data results. Therefore, it is of great significance to use SERS as a new detection method to detect heavy metal ions in water and to characterize the adsorption process of heavy metal ions through the interaction between heavy metals and functional groups.

Here, according to Lewis’s hard and soft acid-base theory, the sulfhydryl group has a strong affinity for heavy metal ions, and it can capture heavy metal ions in the form of coordination in water by forming chelates. Nano titanium dioxide (nano TiO_2_) has a large specific surface area, resulting in high surface activity and a large number of suspension bonds. The Ti and O atoms on the surface of the particles can easily adsorb OH^−^ and H^+^ in the water to form surface titanium ions rich in -OH, which can make the grafting reaction proceed smoothly. In order to realize organic-inorganic hybrid nanomaterials as a heavy metal ion adsorption material, we demonstrate the use of MPTMS containing -SH to modify the nano titanium dioxide to obtain a new nano adsorption material. A series of single-factor parallel experiments were carried out to determine the optimal adsorption conditions, and the maximum adsorption efficiency of nano TiO_2_-SH for the Hg^2+^, Cd^2+^,Pb^2+^ three heavy metal ions reached 98.3%, 98.4% and 98.4% respectively. And more importantly, after five cycles of adsorption and desorption, the adsorption efficiency of nano TiO_2_-SH for these three metal ions is still above 96%. We also used Surface-Enhanced Raman Spectroscopy (SERS) to accurately characterize the characteristic peaks of trace relevant groups before and after the adsorption of nano TiO_2_-SH, which is of great significance for the monitoring and removal of heavy metal ions in water.

## Materials and methods

### Materials

Hydrochloric acid, sodium hydroxide, nitric acid, butyl titanate, ethanol were purchased from National Medicine. Mercury nitrate [Hg(NO)_2_ H,O], cadmium nitrate [Cd(NO)_2_·4H,O], Lead chloride [PbCl_2_] were purchased from Macklin. 3-Propyl hydrophobic trimethoxysilane [(OCH_3_)_3_Si (CH_2_)_3_SH] (MPTMS) was purchased from Alfa Aesar Inc.

### Synthesis of nano TiO_2_

5 g of titanium alkyd was dissolved in 20 ml ethanol solution (95%), and 0.5 ml glacial acetic acid (inhibitor) was added to form chelate with butyl titanate, so that butyl titanate was hydrolyzed uniformly, and uniform colloid solution was obtained. After vacuum evaporation and drying at 120°C, the sol was gradually transformed into gel. White TiO_2_ nanomaterials were obtained by calcination of the gel at 250°C for 1 h.

### Synthesis of nano TiO_2_-SH

59.5 ml anhydrous ethanol and 10.5 ml ultrapure water were accurately taken into a 100 ml conical flask, and 600 ul ammonia water and 0.42 g MPTMS were added, and the reaction was carried out in a water bath at 65°C for 3 h. After the reaction, it was removed and left standing for 15 min, and 1 g nano TiO_2_ was slowly added under 30 min of intense agitation. It was stirred in nitrogen atmosphere for 6 h at room temperature. After the reaction, it was alternately washed with ethanol and ultrapure water for 3 times, centrifuged, and vacuum dried at 80°C for 10 h to obtain the sulfur-modified nano titanium dioxide (nano TiO_2_-SH).

### Determine the content of -SH in nano TiO_2_-SH

Ellman method was used to determine the content of -SH in sulfur-based nano TiO_2_ materials. 0.5 g nano-TiO_2_-SH, drop 3 ml ultra-pure water, add 1 ml DTNB (2-nitrobenzoic acid) and 0.3 ml phosphate buffer solution, stand at room temperature for 4 h, centrifuge for 15 min, take the supernatant to measure the absorbance at 412 nm. The -SH content in nano TiO_2_-SH material was calculated.

### Adsorption experimental methods

The adsorption performance of heavy metal ions (Hg^2+^, Cd^2+^, and Pb^2+^) on nano TiO_2_-SH was investigated through intermittent batch experiments. Vials containing these heavy metal ions were placed in a thermostatic water bath vibrator and stirred at a fixed vibration rate of 200 r min^−1^. Simultaneously, under the different pH conditions, the different heavy metal ion concentration, the different temperature and contact time on adsorption were studied. The residual concentrations of heavy metal ions were analyzed using atomic absorption spectrophotometer (AAS) and atomic fluorescence spectroscopy (AFS), and each experiment was repeated more than three times. The equilibrium adsorption capacity (q_e_) and removal rate (R_e_) of the three metal ions on nano TiO_2_-SH were calculated using the following equations: (Choi et al., 2020; Shi et al., 2022)

Where the C_o_ and C_e_ (mmol L^−1^) are the initial concentration and equilibrium concentration of the testing metal ions, respectively. V (L) is the actual volume of the metal ions solution, and W (g) is the mass of the adsorbent.

### Desorption experiment

In this experiment, HCl solution was used as the eluent to study the relationship between elution efficiency and eluent concentration. The experimental results show that when the concentration of HCl solution is 0.05%–1.5%, the elution efficiency increases with the increase of eluent concentration. In order to ensure the efficient recovery of Hg^2+^, Cd^2+^, and Pb^2+^ ion, 1.5% hydrochloric acid was selected as the eluent in this experiment.

### Measurements and characterization

Attenuated total reflectance-Fourier transform infrared (ATR-FTIR) spectroscopy was measured by a Thermo Scientific Nicolet iS50. Raman spectroscopy was measured by a Thermo Scientific DXR2. The test of solution particle size was carried out on a Malvern Zetasizer AT. Before testing, the solution is first treated with ultrasound to ensure uniform particle dispersion. Thermal stability analysis was conducted on a TA Instruments Q800 thermogravimetric analyzer (TGA) under a nitrogen atmosphere. Each text sample weight control in 3–5 mg and was heated from 25°C to 600°C at a heating rate of 10°C min^−1^. The samples were scan from diffraction angle 10°–80° with a scan speed of 5°/min in x-ray diffraction (XRD) test. The morphologies and microstructures of the as-prepared samples were determined by TESCAN scanning electron microscope, transmission electron microscopy (JEOL).

### PFO and PSO equation

Kinetic models of the linear pseudo-first-order (PFO) and pseudo-second-order (PSO) rate laws were selected. (Choi et al., 2020; Shi et al., 2022).

Where q_e_ and q_t_ (mmol g^−1^) are the adsorption capacity of three metals (Hg^2+^,Cd^2+^,Pb^2+^) at equilibrium and at time t (min), respectively, whereas k_1_ (min^−1^) and k_2_ (g mmol^−1^ min^−1^) are the rate constants of PFO and PSO.

## Results and discussion

**FIGURE 1:**
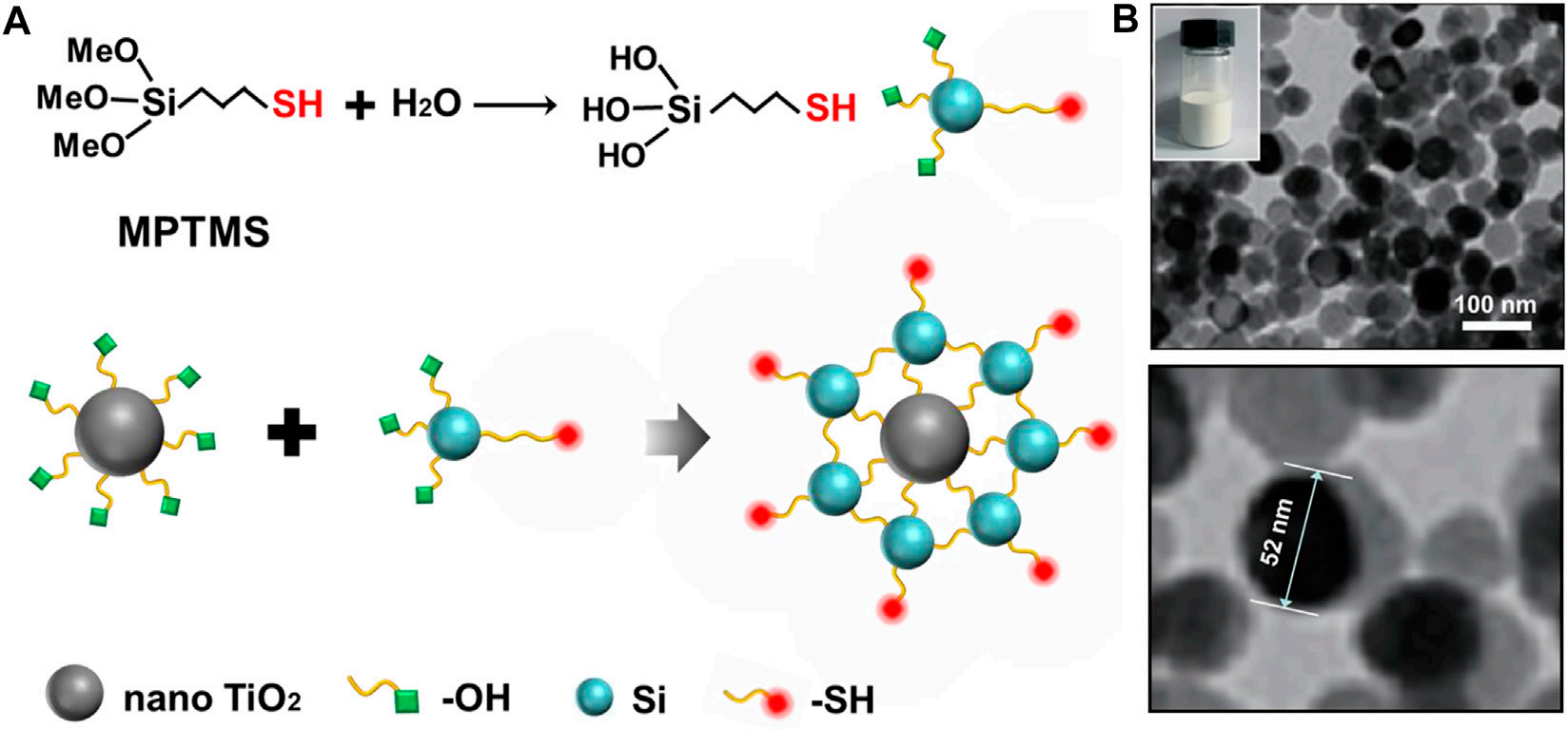
**(A)** The preparation mechanism and structure diagram of nano TiO_2_-SH. **(B)** TEM image of nano TiO_2_-SH, inset is an optical photograph of nano TiO_2_-SH.

In our experiment, nano TiO_2_ was firstly prepared by the sol-gel method (Supplementary Figure S1). The prepared nano TiO_2_ material was characterized by scanning electron microscopy, Fourier infrared, Raman, X-ray diffraction and dynamic light scattering, and the results showed that the nano TiO_2_ material with a particle size of about 50 nm was successfully synthesized. (Supplementary Figure S2). As shown in Figure 1A, MPTMS was first grafted with alcohol hydroxyl groups, and then grafted with the prepared nano TiO_2_ in a certain solvent environment to obtain the -SH modified nano TiO_2_-SH novel organic-inorganic hybrid material. Through TEM characterization of nano TiO_2_-SH, it was found that the prepared nanoparticles were uniform in size and the size was similar to that of the original nano TiO_2_ (52 nm, Figure 1B).

**FIGURE 2:**
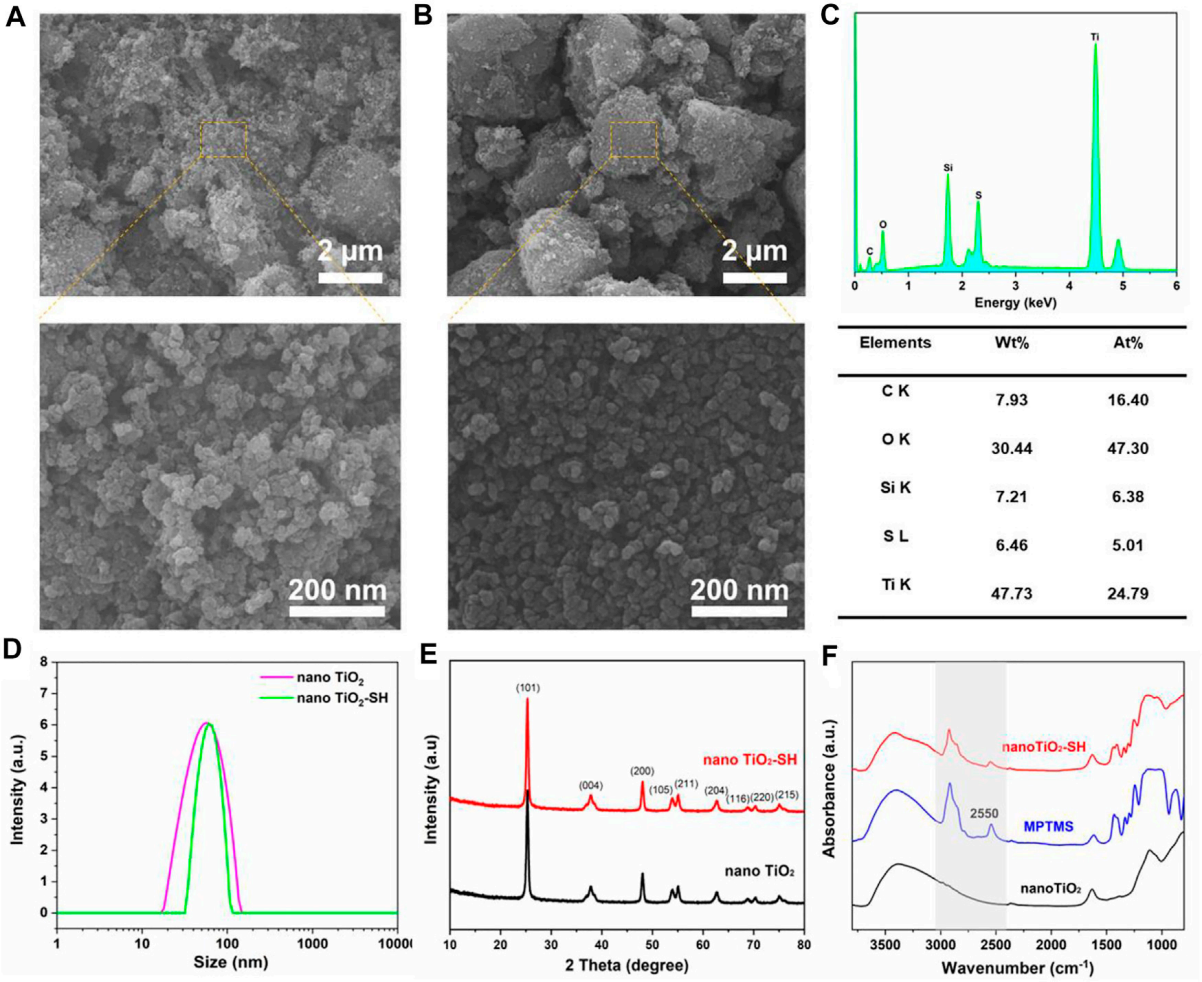
**(A)** SEM image of nano TiO_2_. **(B)** SEM image of nano TiO_2_-SH. **(C)** Content of various elements in nano TiO_2_-SH. **(D)** The particle size distribution of nano TiO_2_ and nano TiO_2_-SH. **(E)** XRD spectra of nano TiO_2_ and nano TiO_2_-SH. **(F)** ATR-FTIR spectra of nano TiO_2_, MPTMS and nano TiO_2_-SH.

Additionally, in order to further prove the success of the modification of nano TiO_2_-SH material, we also carried out comparative tests on the prepared nano TiO_2_ and nano TiO_2_-SH. As shown in Figure 2A, B, it can be clearly seen from the SEM characterization results that the particles of nano TiO_2_ and nano TiO_2_-SH are spherical with almost no difference in size (≈50 nm), but interestingly, the -SH graft-modified nano TiO_2_ showed a better uniform distribution than the ungrafted nano TiO_2_. This was due to the presence of a large amount of -OH on the surface of nano TiO_2_ materials, the hydroxyl groups on the nano TiO2 are clustered by hydrogen bonding interactions, resulting in a certain degree of agglomeration. (Ho et al., 2016). However, since there is almost no -OH on the surface of nano TiO_2_ modified by -SH, the agglomeration phenomenon is significantly reduced, which also ensures that the particle size is more uniform, the boundary is more obvious, and it is more conducive to the dispersion of particles. This result can also be reflected in the DLS test results. In Figure 2D, it can be seen that nano TiO_2_-SH has a narrower particle size distribution than nano TiO_2_, indicating that nano TiO_2_-SH nanoparticles have a more uniform particle size distribution. In addition, the mapping diagram of element distribution on the surface of materials also shows the sulfur element on the surface of nano TiO_2_-SH material and the excellent dispersion and uniformity of each element (Supplementary Figure S3). The elemental analysis spectrogram also proves the existence of the S element and Si element. This proves that MPTMS has been successfully grafted onto nano TiO_2_ (Figure 2C). Moreover, the Tg test curve results illustrate the prepared hybrid nanomaterials have organic components inside, which are carbonized at high temperatures, thereby reducing mass (Supplementary Figure S4).

To further reveal the internal structure of nano TiO_2_-SH, we performed X-ray diffraction (XRD), Fourier transform infrared (FT-IR) spectroscopy and Raman spectroscopy analyses. The XRD crystal peaks showed that the unmodified nano TiO_2_ had three typical characteristic diffraction peaks at 2θ = 25.3°, 38° and 48°, indicating that it was standard anatase type nano TiO_2_, and the three diffraction peaks corresponding to the three crystal planes (101), (004) and (200) in the material respectively. The diffraction peak position and intensity of the modified nano TiO_2_-SH were consistent with that of nano TiO_2_, indicating that the modified titanium dioxide was still standard anatase type, and the crystallinity and morphology were not changed, further indicating that MPTMS only had condensation reaction with the hydroxyl group on the surface of nano TiO_2_ to make -SH access smoothly. It does not change the crystal structure of nano-TiO_2_ (Figure 2E). The FT-IR region at 2550 cm^−1^ in MPTMS is attributed to the stretching of -SH, and this characteristic peak appears in nano TiO_2_-SH, indicating the success of -SH modification. Moreover, the characteristic peaks belonging to -CH_3_ and -CH_2_- at 2924 cm^−1^ and 2858cm^−1^ also appeared in nano TiO_2_-SH, which further confirmed the successful modification of nano TiO_2_ by MPTMS.

**FIGURE 3:**
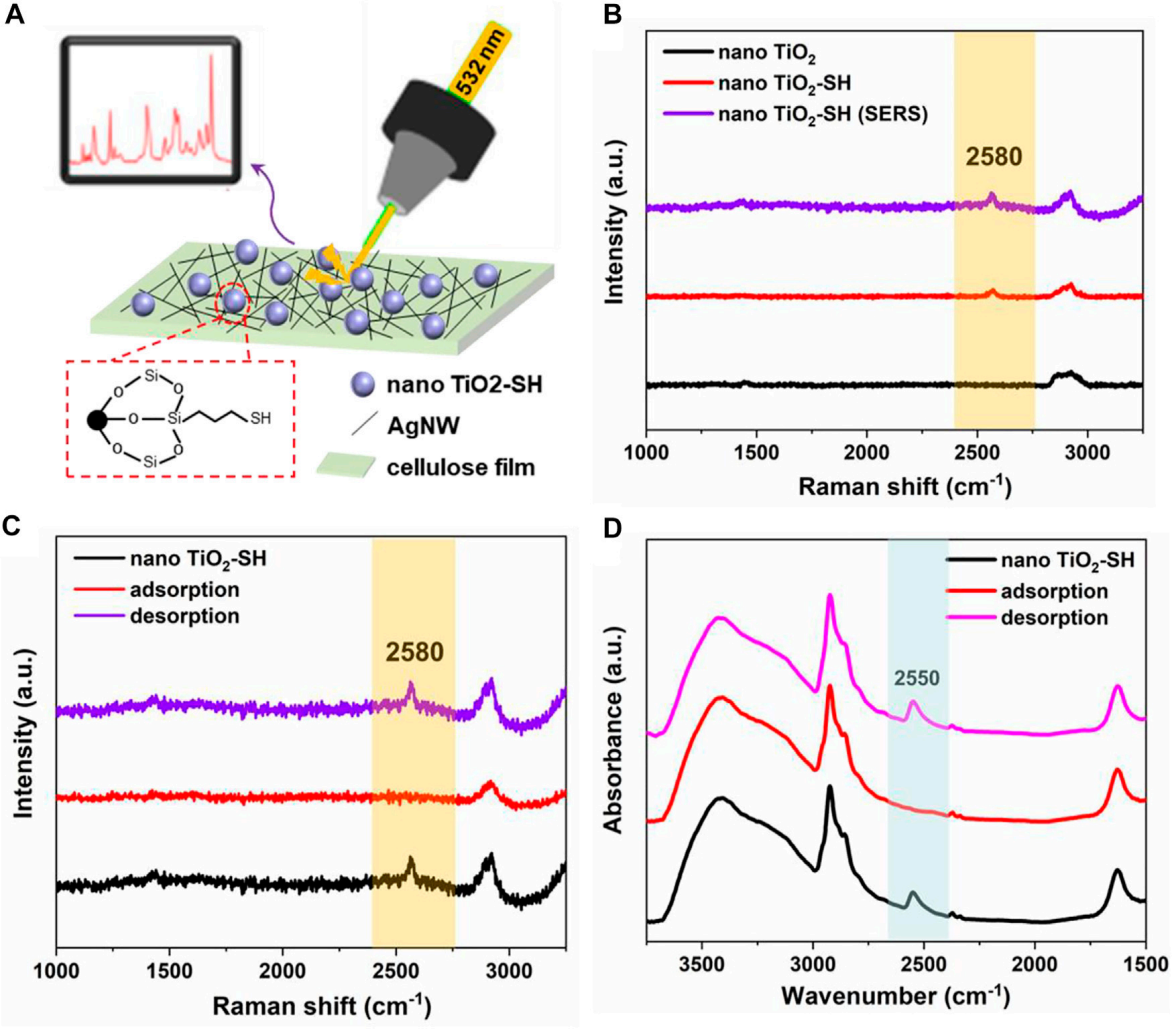
**(A)** The work mechanism and diagram of SERS. **(B)** Raman spectra of nano TiO_2_, nano TiO_2_-SH and nano TiO_2_-SH (SERS). **(C)** SRES of nano TiO_2_-SH before and after adsorption. **(D)** FTIR spectra of nano TiO_2_-SH before and after adsorption.

Surface-enhanced Raman scattering (SERS) is one of the most promising analytical techniques capable of probing a single molecule. When light irradiates the surface of coinage-metal (Au, Ag, and Cu) nanostructures, the surface plasmon resonance (SPR) can be excited, significantly increasing the local electromagnetic field and intensity of Raman scattering. (Cardinal et al., 2017; Grasseschi and Toma, 2017; Sun et al., 2019; Sahin et al., 2022). In our experiment, silver nanowires supported by cellulose film were used as the reinforcement substrate, (Koh et al., 2021; Feng et al., 2022) so as to improve the accurate characterization of related groups of nano TiO_2_-SH hybrid materials and accurate detection of the heavy metal adsorption process of materials in Raman test (Figure 3A). In the Raman test results, the characteristic peaks at 142 cm^−1^, 395 cm^−1^, 513 cm^−1^ and 637 cm^−1^ were corresponding to nano TiO_2_, which proved that nano TiO_2_ was successfully synthesized (Supplementary Figure S5). And as shown in Figure 3B, a characteristic peak attributed to 2580cm^−1^ of -SH appeared in the Raman spectrum of nano TiO_2_-SH, but due to the low content of the -SH group, the peak strength was poor, but through SERS detection, the peak strength was improved, and the occurrence of the group could be identified more clearly. It can be seen that SERS has higher sensitivity for the characterization of the group structure in the material. At the same time, we also carried out SERS characterization of nano TiO_2_-SH before and after heavy metal adsorption, as shown in Figure 3C, the results showed that the -SH characteristic peak in nano TiO_2_-SH disappeared after heavy metal ion adsorption, and the -SH characteristic peak of the material reappeared after desorption and attachment. This is because the chelation of heavy metal and -SH makes the free -SH disappear, and after desorption and attachment, the metal ions leave nano TiO_2_-SH, and the free -SH reappears. Therefore, the characteristic peak of -SH reappears. These phenomena also proved SERS detection method can be used to sensitively and intuitively characterize the adsorption-desorption process of materials for heavy metal ions. Meanwhile, FT-IR was also used to characterize the changes in the -SH group before and after adsorption. The results showed that the characteristic peak at 2550 cm^−1^ attributed to -SH disappeared after adsorption of heavy metal ions, but reappeared after desorption and attachment. This result was also consistent with the detection result of SERS (Figure 3D).

In our experiment, we first explored the influence of the additional amount of MPTMS on -SH content in nano TiO_2_-SH. Ellman method was used to determine the content of -SH in sulfur-based nano-TiO_2_ materials. The results showed that when the amount of MPTMS was less than 0.3 ml, the content of -SH increased with the increase of MPTMS, and when the amount of MPTMS was 0.3 ml, the content of -SH almost did not increase. Therefore, the additional amount of MPTMS was 0.3 ml to prepare nano TiO_2_-SH, and the -SH content in nanomaterials was 3.13 mg/g (Supplementary Figure S6).

**FIGURE 4:**
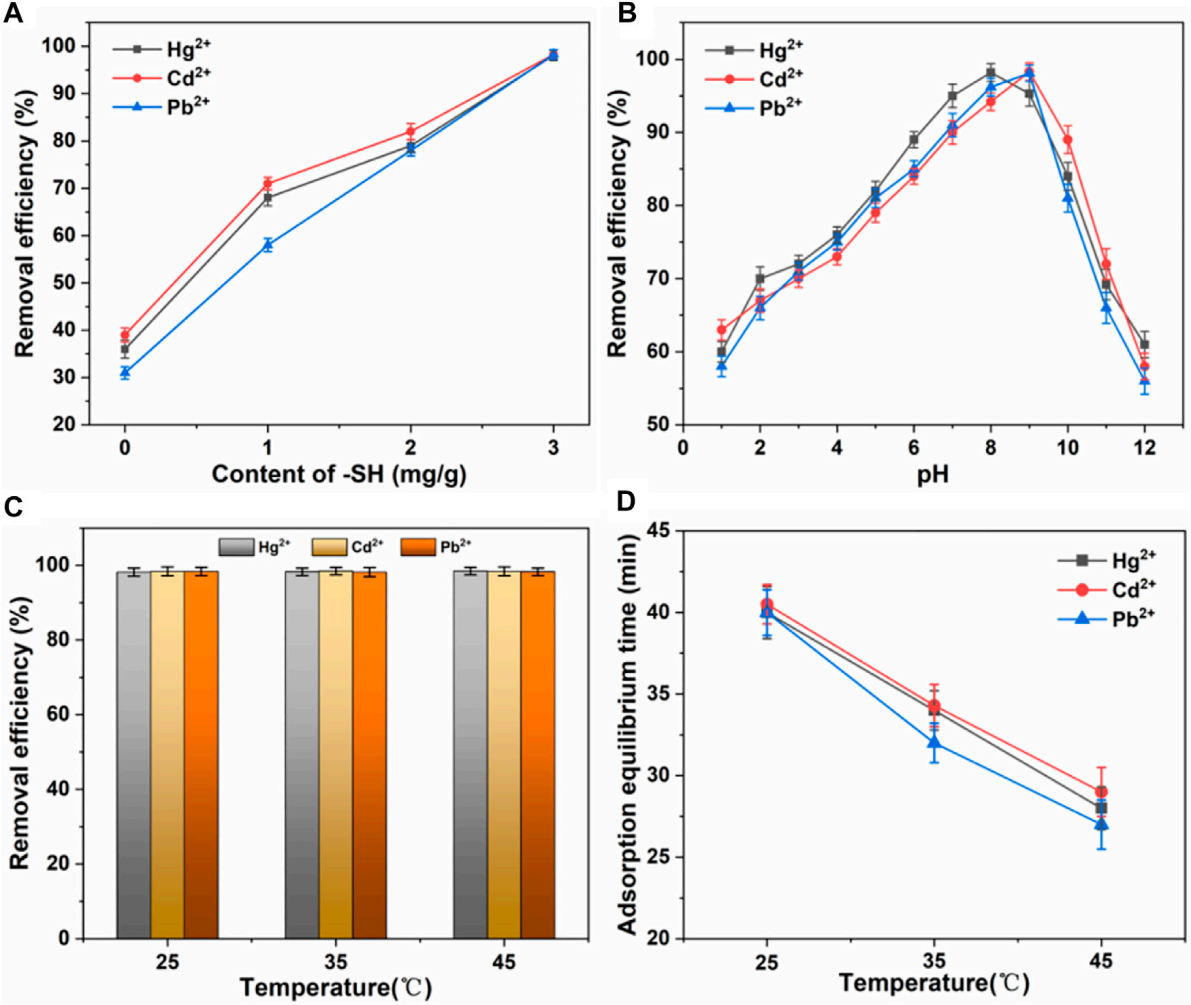
**(A)** Effect of -SH content on adsorption efficiency. **(B)** Effect of pH on adsorption efficiency. **(C,D)** Effect of temperature on adsorption efficiency and equilibrium time.

Next, we use a series of parallel experiments to determine the optimal parameters for material adsorption. (Zhang et al., 2021; Shaheen et al., 2022). First, comparison of the adsorption capacity of nano TiO_2_-SH with different -SH contents for Hg^2+^, Cd^2+^ and Pb^2+^ heavy metal ions was explored, as shown in Figure 4A. The results showed that with the increase of -SH content, the adsorption efficiency of nano TiO_2_-SH for heavy metal ions increased continuously. Finally, the adsorption efficiency of Hg^2+^, Cd^2+^ and Pb^2+^ reached 98.3%, 98.4% and 98.4% respectively. The adsorption efficiency of nano TiO_2_-SH for heavy metal ions also showed great differences under different pH conditions (Figure 4B). Within the range of pH from 1 to 13, the adsorption capacity of nano TiO_2_-SH for Hg^2+^, Cd^2+^ and Pb^2+^ showed a trend of first increasing and then decreasing. In an acid environment, adsorbent adsorption efficiency is low, this may be due to when pH is low, the system in the presence of large amounts of H^+^ will compete and adsorption sites on the surface of the adsorbent, and a large number of H^+^ makes hydrophobic base -SH proton was synthesized on the surface of the nanometer titanium dioxide, reduce -SH content on the surface of the adsorbent, the adsorption capacity is reduced; When pH reaches 8, the adsorption rate of Hg^2+^ reaches 98.3%, and when pH reaches 9, the adsorption rate of Cd^2+^ and Pb^2+^ reaches 98.4%. Subsequently, with the increase of pH value, the solution environment becomes alkaline, and the adsorption rate decreases. This is because when pH is higher, due to the existence of a large number of anions in water, the positively charged metal ions will be surrounded by anions, forming negatively charged atomic groups, affecting the adsorption effect of heavy metal ions. When the pH value of the solution exceeds the precipitation limit of heavy metal ions, a large number of metal ions will exist in the form of hydroxide precipitation, and the Ksp values of the three metal ions are different, so the precipitation rate is also different, which affects the adsorption process. (Li et al., 2015; Wieszczycka et al., 2020). The adsorption temperature has almost no influence on the final adsorption efficiency, as shown in Figure 4C, D, but it greatly affects the adsorption equilibrium time. At a higher temperature, the molecular motion is violent, and the entropy value in the system is high, thus promoting the adsorption process, and the adsorption equilibrium is reached only in about 30 min.

**FIGURE 5:**
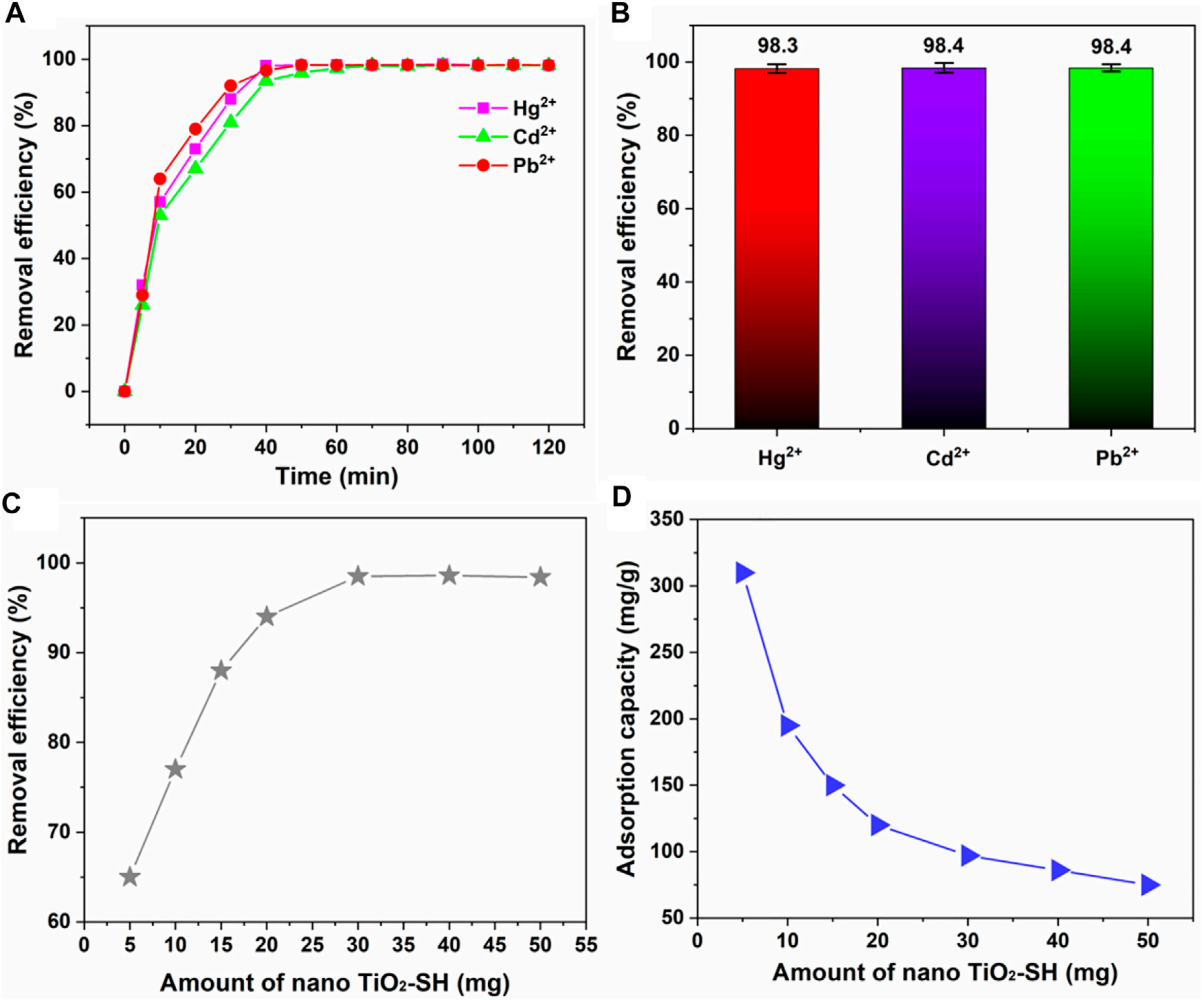
**(A)** The adsorption equilibrium time curve of nano TiO_2_-SH for heavy metal ions (Hg^2+^, Cd^2+^ and Pb^2+^), (25 °C, pH = 8). **(B)** The maximum adsorption efficiency of nano TiO_2_-SH for heavy metal ions. **(C,D)** Influence of the amount of nano TiO_2_-SH on the adsorption efficiency and adsorption capacity of heavy metal ions.

We also evaluated the change curve of the adsorption rate when nano TiO_2_-SH was used as an adsorbent material to adsorb heavy metal ions Hg^2+^, Cd^2+^ and Pb^2+^ at room temperature **(** Figures 5A, B **)**. The results showed that the adsorption efficiency for the three ions increased significantly at the beginning, then slowly increased, and reached the saturation value at about 40min. (98.3%, 98.4%, 98.4%) Then parallel experiments were conducted to compare the amount of adsorbent, as shown in Figures 5C, D. With the increase of the amount of nano TiO_2_-SH, the adsorption efficiency was significantly improved and reached a balance at 30 mg. Subsequently, the adsorption efficiency did not increase with the increase of the amount of adsorbent. This may be due to the increase of the amount of adsorbent, which increases the surface area and active site on the surface of nano TiO_2_-SH, and increases the chance of contact between the adsorption site and heavy metal ions. When the amount of adsorbent continues to increase, the content of heavy metal ions in the system is very low, so the adsorption rate tends to be stable. However, with the increase of the amount of adsorbent, the adsorption capacity of adsorbent decreases gradually, because when the concentration (content) of heavy metal ions is constant, the content of metal ions that can be allocated to the adsorbent per unit mass decreases.

Adsorption kinetics is applied to determine the rate and mechanism of adsorption. To fit the experimental data in this study, kinetic models of the linear pseudo-first-order (PFO) and pseudo-second-order (PSO) rate laws were selected. (Cai et al., 2019; Jiang et al., 2021). As can be seen from Supplementary Figure S7 **,** Supplementary Figure S8, and Supplementary Figure S9 the nano TiO_2_-SH adsorbs heavy metal ions (Hg^2+^, Cd^2+^ and Pb^2+^) at 40 or 50 min when the balance, the level 2 dynamic equation well fitted the experimental results, the regression analysis of the determination coefficient *R* ^2^ value (> 0.999) is close to 1, and the equilibrium adsorption from the calculated through the model’s very close to the experimental results, the adsorption process follows the secondary reaction mechanism. This shows that the adsorption process is mainly chemical adsorption, that is, the chemical adsorption of -SH group on the surface of the material to heavy metal ions. On the other hand, due to the unique characteristics of multi-porosity and the high specific surface area of nanomaterials, there may also be a small amount of physical adsorption to further accelerate the adsorption speed.

**FIGURE 6:**
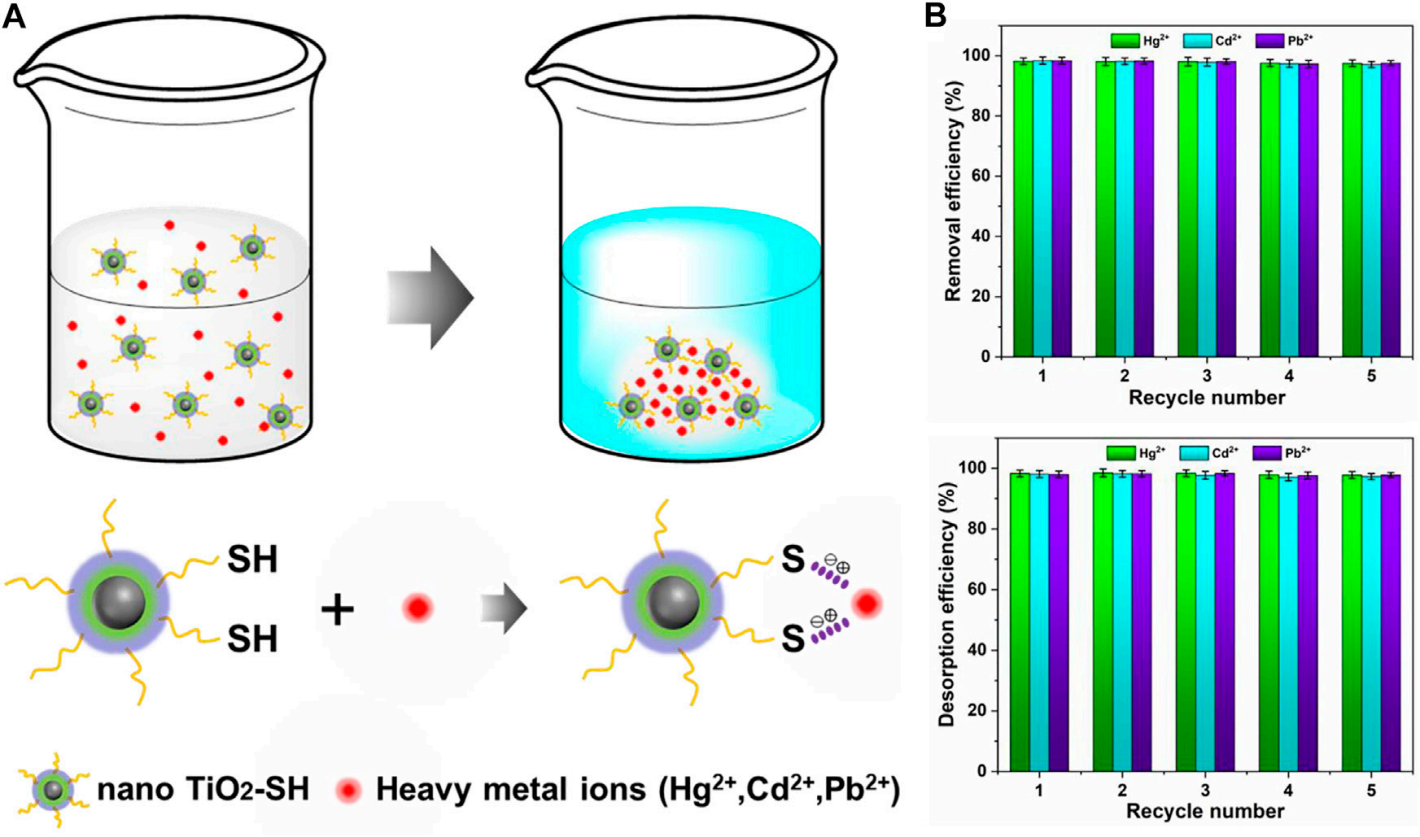
**(A)** Process mechanism of heavy metal ion adsorption by adsorbent materials **(B)** Removal efficiency of adsorption-desorption after five recycle.

Based on the structure character analysis and experimental data results, we give the mechanism of adsorption of heavy metal ions by the nano TiO_2_-SH in Figure 6A. Heavy metal ions (Hg^2+^, Cd^2+^, Pb^2+^) with a positive charge are captured by S^2-^ ions with a negative charge which lose hydrogen atoms and then interact with each other so that the heavy metal ions are firmly blocked to nano TiO_2_-SH so that the heavy metal ions in aqueous solution are removed and the quality of an aqueous solution is improved. According to previous studies, the regeneration and stability of adsorbents are the definitive indicators for the evaluation of their performance for practical industrial applications. (Kamran et al., 2019; Jin et al., 2022). As shown in Figure 6B, the adsorption efficiency of the adsorbent for Hg^2+^, Cd^2+^, and Pb^2+^ reduced from 98.3%, 98.4%, and 98.4%–97.2%, 97.7%, and 97.4% after recycling for 5 times, respectively. Similarly, the desorption efficiency reduced from 97.9%, 97.7%, 98.3%, and 96.2%–96.9%, and 97.1%, respectively. It can be seen that nano TiO_2_-SH, as an adsorbent material for heavy metal ions, still has a very high adsorption efficiency (>96%) for these three metal ions after five cycles of the adsorption-desorption process. In general, the regeneration experiments confirmed that nano TiO_2_-SH as a heavy metal ion adsorbent possesses excellent regeneration and reuse potential.

## Conclusion

In this work, we have successfully fabricated a new type of nano TiO_2_-SH hybrid material, we use MPTMS to modify the nano TiO_2_ to obtain a functional -SH structure which can sensitively capture heavy metal ions. Our system shows excellent adsorption efficiency for a variety of heavy metals (Hg^2+^, Cd^2+^, Pb^2+^), which can greatly expand the application range of this material. The adsorption efficiency of the nano adsorbent for the three heavy metal ions (Hg^2+^, Cd^2+^, Pb^2+^) reached 98.3%, 98.4% and 98.4%, respectively. And after five cycles of adsorption and desorption, the adsorption efficiency of nano TiO_2_-SH for these three metal ions is still above 96%. In addition, we also used Surface-Enhanced Raman Spectroscopy (SERS) to accurately characterize the characteristic peaks of trace relevant groups before and after the adsorption of nano TiO_2_-SH, which is of great significance for the monitoring and removal of heavy metal ions in water. Based on the findings indicating the effective removal of heavy metal ions by nano TiO_2_-SH, we believe this distinct approach will advance the development of the monitoring and treatment of sewage containing heavy metal ions.\

## Data Availability

The original contributions presented in the study are included in the article/Supplementary Material, further inquiries can be directed to the corresponding authors.

## References

[B1] Bolisetty S. Peydayesh M. Mezzenga R. (2019). Sustainable technologies for water purification from heavy metals: Review and analysis. Chem. Soc. Rev. 48, 463–487. 10.1039/c8cs00493e 30603760

[B2] Cai W. Gu M. Jin W. Zhou J. (2019). CTAB-functionalized C@SiO2 double-shelled hollow microspheres with enhanced and selective adsorption performance for Cr(VI). J. Alloy. Compd. 777, 1304–1312. 10.1016/j.jallcom.2018.11.070

[B3] Cardinal F. Ende E. V. Hackler R. A. OmcAnally M. Stair Peter C. Schatz George C. (2017). Expanding applications of SERS through versatile nanomaterials engineering. Chem. Soc. Rev. 46, 3886–3903. 10.1039/c7cs00207f 28640313

[B4] Chen J. Dong R. Chen S. Tang D. Lou X. Ye C. (2022). Selective adsorption towards heavy metal ions on the green synthesized polythiophene/MnO_2_ with a synergetic effect. J. Clean. Prod. 338, 130536. 10.1016/j.jclepro.2022.130536

[B5] Choi H. Y. Bae J. H. Hasegawad Y. Ane S. Kimd I. S. Leea H. (2020). Thiol-functionalized cellulose nanofiber membranes for the effective adsorption of heavy metal ions in water. Carbohydr. Polym. 234, 115881. 10.1016/j.carbpol.2020.115881 32070504

[B6] Dong H. Zhuang Z. Gu Y. Gao J. (2017). The adsorption and activation of formic acid on different anatase TiO_2_ surfaces. J. Energy Chem. 26, 738–742. 10.1016/j.jechem.2017.03.009

[B7] Feng C. Wang X. Yang J. Xi S. Jia M. Shen J. (2022). Silver nanoparticle-decorated chitosan aerogels as threeDimensional porous surface-enhanced Raman scattering substrates for ultrasensitive detection. ACS Appl. Nano Mat. 5, 5398–5406. 10.1021/acsanm.2c00375

[B8] Grasseschi D. Toma H. E. (2017). The SERS effect in coordination chemistry. Coord. Chem. Rev. 333, 108–131. 10.1016/j.ccr.2016.11.019

[B9] Gupta K. Joshi P. Gusain R. Khatri O. P. (2021). Recent advances in adsorptive removal of heavy metal and metalloid ions by metal oxide-based nanomaterials. Coord. Chem. Rev. 445, 214100. 10.1016/j.ccr.2021.214100

[B10] Hasanpour M. Hatami M. (2020). Application of three dimensional porous aerogels as adsorbent for removal of heavy metal ions from water/wastewater: A review study. Adv. Colloid Interface Sci. 284, 102247. 10.1016/j.cis.2020.102247 32916456

[B11] He M. Xu Z. Hou D. Gao B. Cao X. Ok Y. S. (2022). Waste-derived biochar for water pollution control and sustainable development. Nat. Rev. Earth Env. 3, 444–460. 10.1038/s43017-022-00306-8

[B12] Ho T. Chang S. Li C. (2016). Effect of surface hydroxyl groups on the dispersion of ceramic powders. Mat. Chem. Phys. 172, 1–5. 10.1016/j.matchemphys.2016.01.060

[B13] Huang R. Zhang S. Ding J. Meng Y. Zhong Q. Kong D. (2019). Effect of adsorption properties of phosphorus-doped TiO_2_ nanotubes on photocatalytic NO removal. J. Colloid Interface Sci. 553, 647–654. 10.1016/j.jcis.2019.06.063 31252180

[B14] Jiang X. Pan W. Xiong Z. Zhang Y. Zhao L. (2021). Facile synthesis of layer-by-layer decorated graphene oxide based magnetic nanocomposites for β-agonists/dyes adsorption removal and bacterial inactivation in wastewater. J. Alloy. Compd. 870, 159414. 10.1016/j.jallcom.2021.159414

[B15] Jin Y. Liu Z. Han L. Zhang Y. Li L. Zhu S. (2022). Synthesis of coal-analcime composite from coal gangue and its adsorption performance on heavy metal ions. J. Hazard. Mat. 423, 127027. 10.1016/j.jhazmat.2021.127027 34481383

[B16] Kamran U. Heo Y. Lee J. W. Park S. (2019). Chemically modified activated carbon decorated with MnO2 nanocomposites for improving lithium adsorption and recovery from aqueous media. J. Alloy. Compd. 794, 425–434. 10.1016/j.jallcom.2019.04.211

[B17] Koh E. H. Lee W. Choi Y. Moon J. Jang J. Park S. (2021). A wearable surface-enhanced Raman scattering sensor for labelfree molecular detection. ACS Appl. Mat. Interfaces 13, 3024–3032. 10.1021/acsami.0c18892 33404230

[B18] Li R. Wu M. Shi Y. Aleid S. Wang W. Zhang C. (2021). Hybrid water vapor sorbent design with pollution shielding properties: Extracting clean water from polluted bulk water sources. J. Mat. Chem. A 9, 14731–14740. 10.1039/d1ta03543f

[B19] Li X. Zhou H. Wu W. Wei S. Xu Y. Kuang Y. (2015). Studies of heavy metal ion adsorption on Chitosan/Sulfydryl-functionalized graphene oxide composites. J. Colloid. Interface Sci. 448, 389–397. 10.1016/j.jcis.2015.02.039 25746192

[B20] Ma D. Yang S. Hao X. (2020). Graphene oxide-montmorillonite/sodium alginate aerogel beads for selective adsorption of methylene blue in wastewater. J. Alloy. Compd. 832, 154833. 10.1016/j.jallcom.2020.154833

[B21] Ma T. Sun S. Fu G. Hall J. W. Ni Y. He L. (2020). Pollution exacerbates China's water scarcity and its regional inequality. Nat. Commun. 11, 650. 10.1038/s41467-020-14532-5 32005847 PMC6994511

[B22] Qiang T. Song Y. Zhu R. Yuan W. (2020). Biomass material derived hierarchical porous TiO_2_: Adjustable pore size for protein adsorption. J. Alloy. Compd. 829, 154512. 10.1016/j.jallcom.2020.154512

[B23] Roy J. (2022). The synthesis and applications of TiO_2_ nanoparticles derived from phytochemical sources. J. Ind. Eng. Chem. 106, 1–19. 10.1016/j.jiec.2021.10.024

[B24] Sahin F. Celik N. Ceylan A. Pekdemir S. Ruzi M. Serdar Onses M. (2022). Antifouling superhydrophobic surfaces with bactericidal and SERS activity. Chem. Eng. J. 431, 133445. 10.1016/j.cej.2021.133445

[B25] Shaheen A. AlBadi S. Zhuman B. Taher H. Banat F. AlMarzooqi F. (2022). Photothermal air gap membrane distillation for the removal of heavy metal ions from wastewater. Chem. Eng. J. 431, 133909. 10.1016/j.cej.2021.133909

[B26] Shao Y. Wang Y. Zhu D. Xiong X. Tian Z. Balakin A. V. (2022). Measuring heavy metal ions in water using nature existed Microalgae as medium based on Terahertz technology. J. Hazard. Mat. 435, 129028. 10.1016/j.jhazmat.2022.129028 35525009

[B27] Shi T. Xie Z. Zhu Z. Shi W. Liu Y. Liu M. (2022). Highly efficient and selective adsorption of heavy metal ions by hydrazide-modified sodium alginate. Carbohydr. Polym. 276, 118797. 10.1016/j.carbpol.2021.118797 34823803

[B28] Singha S. Kaura P. Aggarwala D. Kumarb V. Tikoob K. Bansalc S. (2022). Polyaniline enwrapped CoFe_2_O_4_/g-CN ternary nanocomposite for adsorption driven photocatalytic degradation of explicitly diverse organic pollutants. J. Alloy. Compd. 923, 166255. 10.1016/j.jallcom.2022.166255

[B29] Sun H. Cong S. Zheng Z. Wang Z. Chen Z. Zhao Z. (2019). Metal–organic frameworks as surface enhanced Raman scattering substrates with high tailorability. J. Am. Chem. Soc. 141, 870–878. 10.1021/jacs.8b09414 30566339

[B30] Wang H. Wang Z. Yue R. Gao F. Ren R. Wei J. (2020). Rapid preparation of adsorbent based on mussel inspired chemistry and simultaneous removal of heavy metal ions in water. Chem. Eng. J. 383, 123107. 10.1016/j.cej.2019.123107

[B31] Wieszczycka K. Filipowiak K. Wojciechowska I. Aksamitowski P. (2020). Novel ionic liquid-modified polymers for highly effective adsorption of heavy metals ions. Sep. Purif. Technol. 236, 116313. 10.1016/j.seppur.2019.116313

[B32] Wu T. Liu C. Kong B. Sun J. Gong Y. Li K. (2019). Amidoxime-Functionalized macroporous carbon self-refreshed electrode materials for rapid and high-capacity removal of heavy metal from water. ACS Cent. Sci. 5, 719–726. 10.1021/acscentsci.9b00130 31041392 PMC6487541

[B33] Xie H. Zhang S. Zhong L. Wang Q. Hu J. Tang A. (2021). Effect of the occurrence state of magnesium in talc on the adsorption of Pb(II). J. Alloy. Compd. 887, 161288. 10.1016/j.jallcom.2021.161288

[B34] Xu C. Feng Y. Li H. Wu R. Ju J. Liu S. (2022). Adsorption of heavy metal ions by iron tailings: Behavior, mechanism, evaluation and new perspectives. J. Clean. Prod. 344, 131065. 10.1016/j.jclepro.2022.131065

[B35] Yan Z. Chen Y. Bao X. Zhang X. Ling X. Lu G. (2021). Microplastic pollution in an urbanized river affected by water diversion: Combining with active biomonitoring. J. Hazard. Mat. 417, 126058. 10.1016/j.jhazmat.2021.126058 34015710

[B36] Zhang Y. Xu G. Xu M. Wang D. Wang H. Zhan Y. (2021). Preparation of MgO porous nanoplates modified pumice and its adsorption performance on fluoride removal. J. Alloy. Compd. 884, 160953. 10.1016/j.jallcom.2021.160953

[B37] Zhou Z. Yu Y. Ding Z. Zuo M. Jing C. (2019). Competitive adsorption of arsenic and fluoride on TiO_2_ . Appl. Surf. Sci. 466, 425–432. 10.1016/j.apsusc.2018.10.052

