# Supplementary figures and images for: Effective adsorption of heavy metal ions in water by sulfhydryl modified nano titanium dioxide
Source: Front Chem. 2023 Jan 27;10:1072139. doi: 10.3389/fchem.2022.1072139 (PMC9911413; doi:10.3389/fchem.2022.1072139)

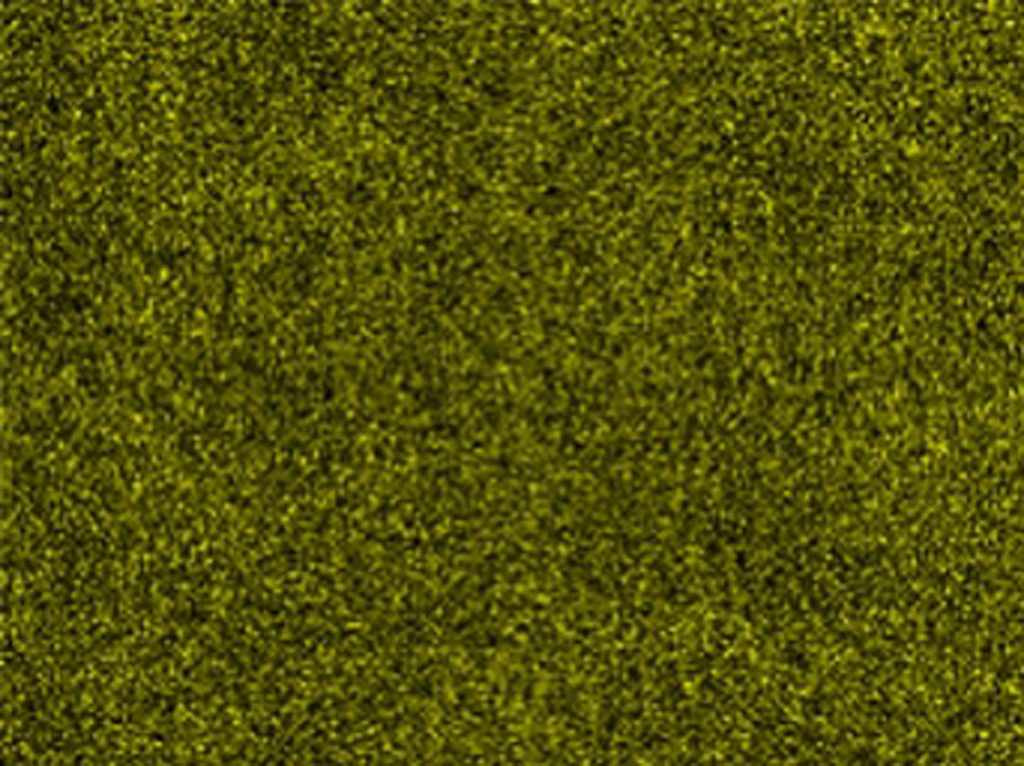

Supplement: Supplementary file 1 [file DataSheet1.zip › Original data/Fig.S3-1.jpg]

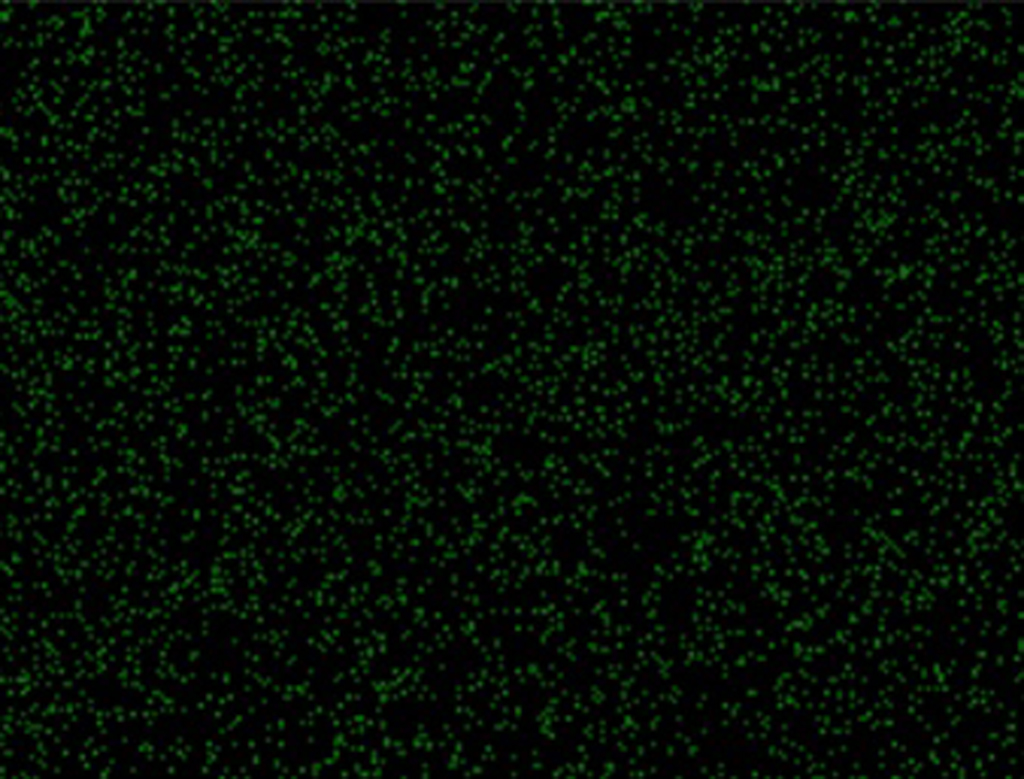

Supplement: Supplementary file 1 [file DataSheet1.zip › Original data/Fig.S3-2.jpg]

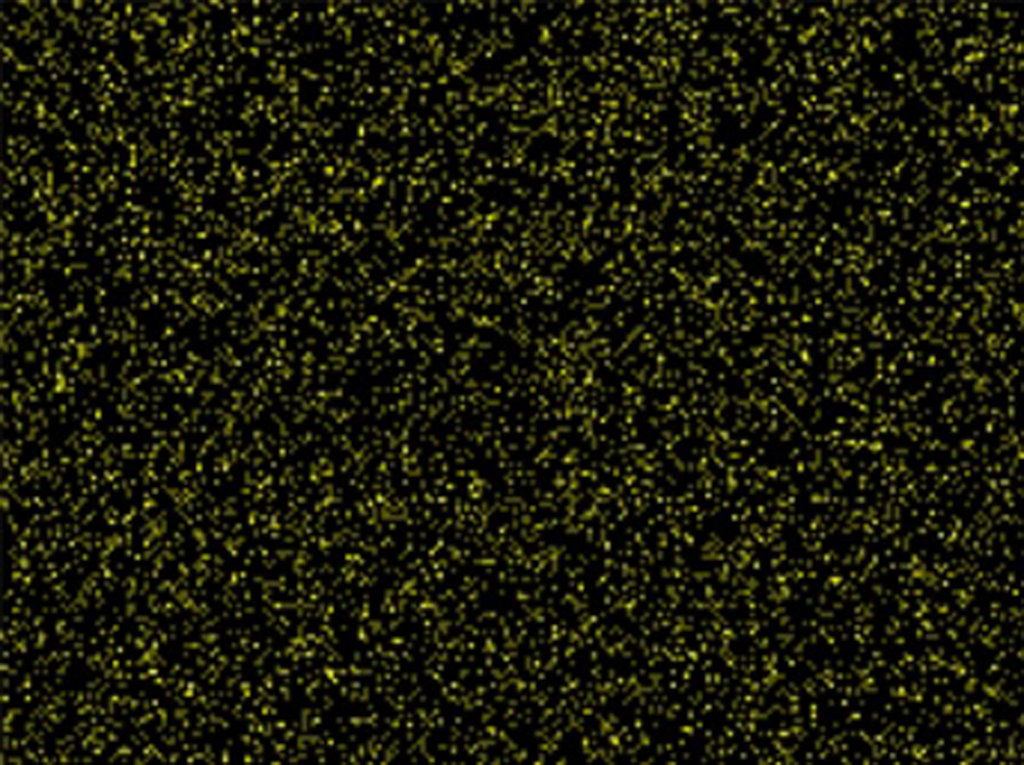

Supplement: Supplementary file 1 [file DataSheet1.zip › Original data/Fig.S3-3.jpg]

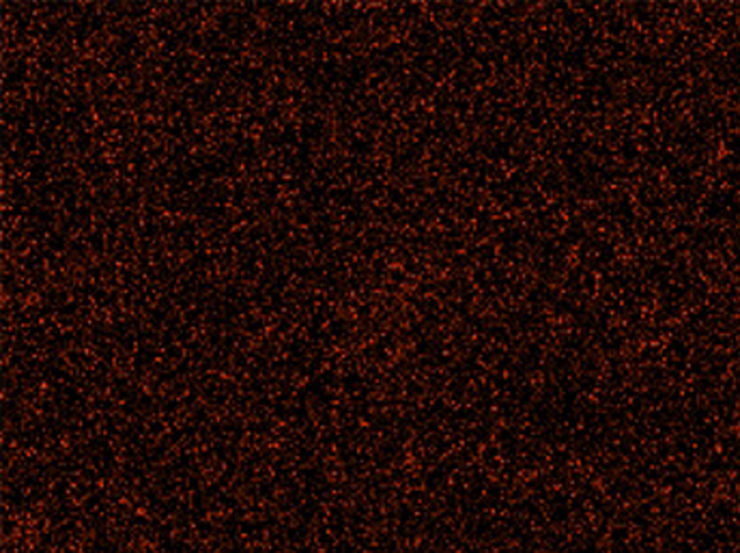

Supplement: Supplementary file 1 [file DataSheet1.zip › Original data/Fig.S3-4.jpg]
